# Angiotensinogen M235T Gene Polymorphism and Risk of Ischemic Heart Disease Complication among Patients with Hypertension in the Ethiopian Population

**DOI:** 10.4314/ejhs.v35i3.3

**Published:** 2025-05

**Authors:** Addisu Melake, Mihretu Jegnie

**Affiliations:** 1 Biomedical Science Education and Service Directorate, College of Health Science, Debre Tabor University, Debre Tabor, Ethiopia

**Keywords:** angiotensinogen, blood pressure, ischemic heart disease, M235T gene polymorphism, hyperlipidemia

## Abstract

**Background:**

The relationship between angiotensinogen gene variations and other risk factors in ischemic heart disease (IHD) remains unclear, largely due to the complex interplay of genetic and environmental factors. This study aimed to investigate the association of high cholesterol levels, and the angiotensinogen M235T (rs699) gene variant with ischemic heart disease in hypertensive patients.

**Methods:**

A hospital-based case-control study was conducted with 70 hypertensive patients diagnosed with IHD and 70 age- and sex-matched healthy controls. Sociodemographic and clinical data were collected, and blood samples were taken for biochemical and genetic testing. Polymerase chain reaction (PCR), restriction fragment length polymorphism (RFLP), and agarose gel electrophoresis were used to determine the angiotensinogen M235T genotypes.

**Results:**

The AGT-TT genotype (OR = 3.35, 95% CI = 1.30–6.63; P < 0.05) and T allele (OR = 2.50, 95% CI = 1.51–4.14; P < 0.001) were significantly more frequent in patients than in controls. Furthermore, dyslipidemia was more prevalent in the patient group compared to the controls (OR = 4.57, 95% CI = 1.71–12.18; P = 0.0024).

**Conclusion:**

The AGT M235T TT genotype and T allele are associated with ischemic heart disease in hypertensive patients, which may suggest as a potential biomarker for early detection and prevention. Dyslipidemia was higher in ischemic heart disease patients with hypertension.

## Introduction

Ischemic heart disease (IHD), which involves the buildup of fat in the blood vessels, narrowing of arterial passages, and reduced blood flow, eventually leading to ischemic heart failure, is a leading global cause of mortality (1). Atherosclerosis is now understood as a complex condition influenced by various risk factors such as age, sex, smoking, dyslipidemia, hypertension (HTN), and diabetes.

Genetic factors are also recognized as contributing significantly to the progression of IHD (2). Hypertension, a major IHD risk factor, is responsible for 7.5 million deaths annually worldwide. Studies have shown that blood pressure levels are strongly and positively correlated with the progression of ischemic heart disease (3).

Lipids and lipoproteins are increasingly important in clinical practice due to their association with IHD. Dyslipidemia is characterized by elevated blood levels of triglycerides (TG), low-density lipoprotein cholesterol (LDL-C), and total cholesterol (TC), and decreased high-density lipoprotein cholesterol (HDL-C) levels (4). It is estimated that more than 50% of the global population is affected by dyslipidemia, though its prevalence varies by population (5).

Angiotensinogen (AGT), a protein produced by the liver, interacts with renin to form angiotensin I, which is converted into angiotensin II. Angiotensin II plays a key role in the development of IHD by causing vasoconstriction of coronary arteries, stimulating the sympathetic nervous system, and promoting fibroblast proliferation (7). The M235T polymorphism of the AGT gene refers to the substitution of threonine (T) for methionine (M) at position 235, resulting in three genotypes: MM, MT, and TT (8).

Although the AGT M235T gene polymorphism has been extensively studied in relation to cardiovascular diseases, the results have been inconsistent (9). In Ethiopia, while several studies have assessed the prevalence, risk factors, and outcomes of IHD (10, 11), no research has explored the impact of the AGT gene polymorphism on disease development. Therefore, this study aims to investigate the association between the AGT M235T gene polymorphism and hypertensive IHD complications and to evaluate the role of dyslipidemia in the development of IHD in the Ethiopian population.

## Methods and Materials

**Study participants**: A hospital-based case-control study was conducted at Debre Tabor Comprehensive Specialized Hospital between January and April 2023. The hospital provides care and follow-up for severe chronic conditions such as IHD and HTN. The source population consisted of all patients receiving follow-up care at the chronic follow-up clinic (CFC). The study included 70 hypertensive patients with IHD complications, confirmed by electrocardiogram (ECG), and 70 age- and sex-matched healthy controls.

**Inclusion and exclusion criteria**: Hypertensive patients diagnosed with IHD complications through ECG and receiving follow-up care at the CFC for at least one year were included in the study. Controls were normotensive, healthy individuals from the same geographical area, with normal ECG results. Patients with hepatic or renal diseases, secondary hypertension, other cardiac complications, stroke, chronic infections, or those unable or unwilling to provide informed consent were excluded.

**Sample size determination and sampling technique**: The sample size was calculated using G*Power software, with an alpha of 0.05, power (1-β) of 0.8, and an effect size (d) of 0.5, resulting in a total of 140 participants, equally divided between cases and controls. Participants were selected using simple random sampling with a random number table.

**Data collection methods**: Data on socio-demographic and behavioral factors were collected using a semi-structured questionnaire. Body weight and height were measured using portable digital scales and stadiometers, respectively, and body mass index (BMI) was calculated. Blood pressure measurements were taken after five minutes of rest using a digital sphygmomanometer, with the mean of three readings used for analysis.

**Sample collection and laboratory methods**: Five milliliters of blood were drawn from each participant for biochemical and genetic testing. Serum was used for the analysis of total cholesterol (TC), triglycerides (TG), LDL-C, HDL-C, glucose, and creatinine using the Dimension EXL 200 fully automated analyzer. Dyslipidemia was defined as TC >200 mg/dl, TG >150 mg/dl, LDL >130 mg/dl, and HDL <60 mg/dl.

**Figure 1:**
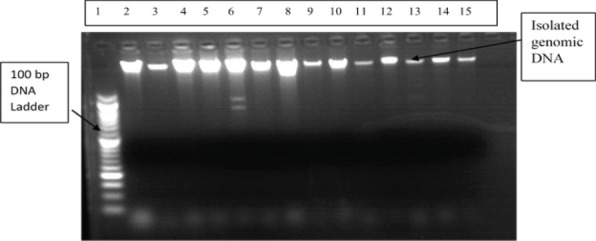
Agarose gel (1%) electrophoresis showing the quality of isolated genomic DNA (Lane 1: 100 bp DNA ladder; Lanes 2 – 15: isolated genomic DNA)

**Figure 2:**
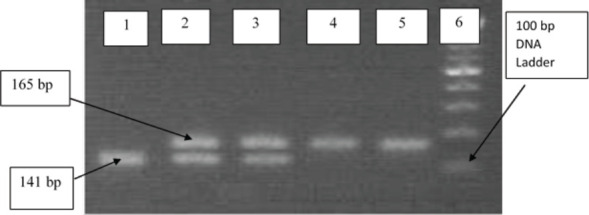
Representative 2% agarose gel electrophoresis showing PCR products of the AGT M235T gene. (Lanes 4 and 5: homozygous TT genotypes; Lanes 2 and 3: heterozygous MT genotypes; Lane 1: homozygous MM genotypes; Lane 6: 100 bp DNA ladder)

**Genomic DNA extraction and genotyping genomic**: DNA was extracted using the salting-out method. AGT M235T genotyping was performed using PCR amplification with specific primers and enzymatic digestion with PsyI restriction enzyme. The PCR products were analyzed by agarose gel electrophoresis (Figure 1) and (Figure 2).

**Statistical analysis**: Data were analyzed using STATA Version 14. Continuous variables were compared using t-tests, and genotype frequencies were compared using chi-square tests. Logistic regression was used to assess the relationship between AGT M235T genotypes and hypertensive IHD. A p-value of <0.05 was considered statistically significant.

## Results

**Socio-demographic and clinical characteristics**: The socio-demographic characteristics of the IHD patient group and the healthy controls were comparable in terms of age and sex distribution. The average age of the IHD patients was 59.1 ± 13.8 years, while the control group had an average age of 57.1 ± 7.1 years. Significant differences were observed in blood pressure, cholesterol levels, and lipid profiles between patients and controls, with IHD patients exhibiting higher systolic and diastolic blood pressure, TC, TG, and LDL-C levels, and lower HDL-C levels (p < 0.001) (Table 1).

**Table 1:** Demographic and clinical characteristics of the study participants in Debre Tabor Referral Hospital, Northwest Ethiopia, 2022

Variables	IHD (n=70)	Control (n=70)	P-value
Sex (M/F)	35/35	37/33	0.7352
Age (yr)	59.84 ± 14.25	57.21 ± 6.66	0.1645
BMI (Kg/m^2^)	23.20 ± 4.22	22.44 ± 4.22	0.2893
SBP (mmHg)	146.62±7.04	116.18±4.04	< 0.001*
DBP (mmHg)	91.08±4.05	75.17±7.75	< 0.001*
FBS (mg/dl)	94.14±18.47	91.02±8.97	0.2066
Creatinine (mg/dl)	0.82±0.15	0.79±0.13	0.3434
Total Cholesterol (mg/dl)	195.90±64.78	149.48±53.34	< 0.001*
Triglyceride (mg/dl)	145.77±74.77	108.40±37.77	< 0.001*
LDL-Cholesterol (mg/dl)	96.97±37.34	75.61±26.82	< 0.001*
HDL-Cholesterol (mg/dl)	43.94±11.31	50.22±9.04	< 0.001*
Dyslipidemia (%)	34.28 %	10.00 %	< 0.001*
Family history of HTN (%)	47.14 %	58.57 %	0.1756
Family history of IHD (%)	14.29 %	21.14 %	0.1423
Smoking habit (yes/no)	12/58	7/63	0.2173
Alcohol intake (yes/no)	44/26	40/30	0.4902
Salt intake (yes/no)	64/6	67/3	0.3012
Physical exercise (yes/no)	5/65	11/59	0.1110
Stress (yes/no)	41/29	34/36	0.2355

* Statistically significant differences at *P* <0.05

**Figure 3:**
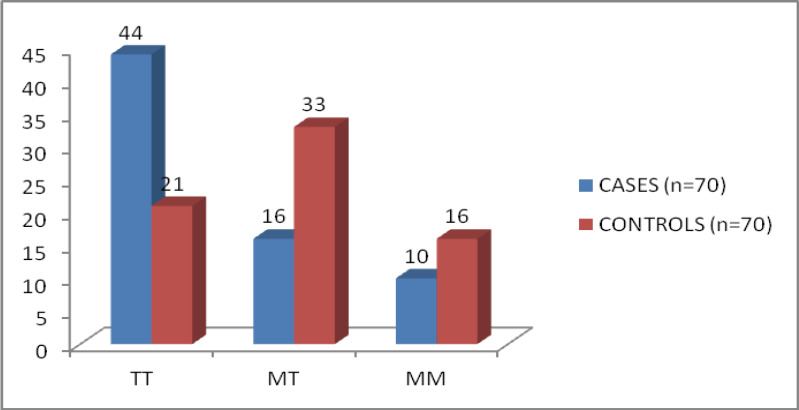
Distribution of the AGT M235T genotype in cases and controls

**Distribution of AGT genotypes and Allele frequencies**: The frequency of the AGT M235T genotypes was significantly different between IHD patients and controls, with the TT genotype more prevalent in patients (OR = 3.35, 95% CI = 1.30–6.63; P < 0.05) than controls. The frequency of the T allele was also higher in patients (OR = 2.50, 95% CI = 1.51–4.14; P < 0.001) than controls (Table 2) and (Figure 3).

**Table 2:** Distribution of AGT genotypes and allele frequencies of the study participants in Debre Tabor Referral Hospital, Northwest Ethiopia, 2022

Variable	IHD (n=70)	Control (n=70)	OR (95% CL)	p-value
**Genotype**				
TT	44 (62.86 %)	21 (30.00 %)	3.35 (1.30 – 6.63)	0.012*
MT	16 (22.86 %)	33 (47.14 %)	0.77 (0.28 – 2.08)	0.615
MM	10 (14.29 %)	16 (22.86 %)	Ref	
**Allele Frequency**				
T	104 (74.29 %)	75 (53.57 %)	2.50 (1.51 – 4.14)	< 0.001*
M	36 (25.71 %)	65 (46.43 %)	Ref	

* Statistically significant differences at *P* <0.05

**Relationship between clinical parameters and AGT genotypes**: No significant correlations were found between AGT genotypes and clinical parameters such as lipid profiles, blood pressure, or fasting blood glucose (P > 0.05) (Table 3).

**Table 3:** Association of AGT M235T genotype with clinical characteristics in Debre Tabor Referral Hospital, Northwest Ethiopia, 2022

Variables	Genotypes			

	TT (n=65)	MT (n=49)	MM (N=26)	p-value
Sex (M/F)	30/28	30/15	16/21	0.3818
Age (yr)	57.72±12.31	59.88±10.69	58.13±9.89	0.5209
Family history HTN (%)	49.2 %	61.2 %	46.1 %	0.8916
Family history IHD (%)	16.9 %	22.4 %	11.5 %	0.7646
BMI (Kg/m^2^)	22.6±4.2	22.6±4.1	23.8±4.5	0.5085
SBP (mmHg)	139.2±17.6	124.7±10.8	124.6±12.9	< 0.01*
DBP (mmHg)	87.1±9.7	79.3±10.6	80.3±7.1	< 0.01*
FBS (mg/dl)	87.0±19.2	89.7±6.3	86.8±7.2	0.2061
TC (mg/dl)	171.8±58.7	169.7±63.5	180.5±76.3	0.1066
TG (mg/dl)	124.2±61.0	124.7±55.9	138.9±75.0	0.3577
LDL-C (mg/dl)	85.8±33.3	84.2±33.2	91.6±38.5	0.6047
HDL-C (mg/dl)	47.2±10.3	46.8±11.4	47.2±10.4	0.5045
Creatinine (mg/dl)	0.83±0.13	0.78±0.15	0.80±0.13	0.3857

* Statistically significant differences at *P* <0.05.

**Association between ischemic heart disease and dyslipidemia**: Dyslipidemia was more common in hypertensive IHD patients (30%) compared to controls (8.57%), with significant differences in lipid profiles observed between the two groups (P < 0.001) (Table 3).

Dyslipidaemia was 4.69 times more likely in individuals with IHD compared to those without (OR = 4.69, 95% CI = 1.86–11.82; P < 0.01) (Table 3).

## Discussion

Ischemic heart disease is influenced by both genetic and environmental factors. In this study, we found a significant association between the AGT M235T polymorphism and increased risk of hypertensive IHD. These findings are consistent with studies in other populations (23-27), but they contrast with research from Saudi Arabia, Caucasian, and Taiwanese populations (28-30). The AGT M235T polymorphism may contribute to IHD risk by increasing angiotensinogen levels, enhancing the renin-angiotensin-aldosterone system (RAAS), and promoting endothelial dysfunction and vascular remodeling (31).

While dyslipidemia was a significant risk factor for IHD in our study, no direct association was found between the AGT gene polymorphism and dyslipidemia, suggesting that other genetic or environmental factors may play a role.

The limitations of this study include the small sample size and the inability to isolate the effect of the M235T polymorphism in hypertensive patients without IHD. Future studies with larger sample sizes and more detailed genetic analysis are needed to further explore these associations.

In conclusion, this study highlights the association between the AGT M235T polymorphism and hypertensive IHD, and may suggest its potential as a biomarker for early detection and prevention. Dyslipidemia, along with high blood pressure, was higher in ischemic heart disease patients. Further research is required to fully understand the genetic underpinnings of IHD and its complications in diverse populations.
